# Estimating the incidence of rotavirus infection in children from India and Malawi from serial anti-rotavirus IgA titres

**DOI:** 10.1371/journal.pone.0190256

**Published:** 2017-12-29

**Authors:** Aisleen Bennett, Nico Nagelkerke, Ellen Heinsbroek, Prasanna S. Premkumar, Małgorzata Wnęk, Gagandeep Kang, Neil French, Nigel A. Cunliffe, Naor Bar-Zeev, Ben Lopman, Miren Iturriza-Gomara

**Affiliations:** 1 Malawi-Liverpool-Wellcome Trust Clinical Research Programme, College of Medicine, University of Malawi, Blantyre, Malawi; 2 Centre for Global Vaccine Research, Institute of Infection & Global Health, University of Liverpool, Liverpool, United Kingdom; 3 Division of Gastrointestinal Sciences, Christian Medical College, Vellore, India; 4 Malawi Epidemiology and Intervention Research Unit/London School of Hygiene & Tropical Medicine, Chilumba, Malawi; 5 Department of Epidemiology, Rollins School of Public Health, Emory University, Atlanta, United States of America; 6 NIHR Health Protection Research Unit in Gastrointestinal Infections, University of Liverpool, Liverpool, United Kingdom; University of North Carolina at Chapel Hill School of Medicine, UNITED STATES

## Abstract

Accurate estimates of rotavirus incidence in infants are crucial given disparities in rotavirus vaccine effectiveness from low-income settings. Sero-surveys are a pragmatic means of estimating incidence however serological data is prone to misclassification. This study used mixture models to estimate incidence of rotavirus infection from anti-rotavirus immunoglobulin A (IgA) titres in infants from Vellore, India, and Karonga, Malawi. IgA titres were measured using serum samples collected at 6 month intervals for 36 months from 373 infants from Vellore and 12 months from 66 infants from Karonga. Mixture models (two component Gaussian mixture distributions) were fit to the difference in titres between time points to estimate risk of sero-positivity and derive incidence estimates. A peak incidence of 1.05(95% confidence interval [CI]: 0.64, 1.64) infections per child-year was observed in the first 6 months of life in Vellore. This declined incrementally with each subsequent time interval. Contrastingly in Karonga incidence was greatest in the second 6 months of life (1.41 infections per child year [95% CI: 0.79, 2.29]). This study demonstrates that infants from Vellore experience peak rotavirus incidence earlier than those from Karonga. Identifying such differences in transmission patterns is important in informing vaccine strategy, particularly where vaccine effectiveness is modest.

## Introduction

Prior to wide-spread rotavirus vaccination, rotavirus was responsible for over 450 000 deaths in young children, with 95% of these deaths occurring in low income, GAVI-the Vaccine Alliance (GAVI)-eligible countries[1]. Two live oral vaccines are currently globally licenced for the prevention of severe rotavirus gastroenteritis, a monovalent and a pentavalent formulation. These vaccines are highly efficacious against severe disease in high and middle income countries (vaccine efficacy 85–100%)[2,3], but have proven much less protective in low income countries (LIC) (vaccine efficacy 49%-64%)[4–6]. The reasons for reduced vaccine efficacy in LIC are not fully understood but hypotheses include vaccine interaction with maternal antibody, micronutrient malnutrition, differences in gut microbiome, concomitant oral polio vaccination, and epidemiological differences such as greater force of infection (rate at which susceptible individuals acquire infection[7]) [8–12]. In view of the high rotavirus gastroenteritis morbidity and mortality, the World Health Organisation (WHO) has recommended routine rotavirus vaccination of infants in LIC and rotavirus vaccine has subsequently been introduced into more than 35 GAVI eligible countries[13]. Post-introduction reports of vaccine effectiveness from LIC have been encouraging (60–70%)[14–16], but remain low compared to high-income settings[17,18].

In the context of a high burden of rotavirus disease and reduced vaccine effectiveness in LIC, understanding patterns of exposure is important for public health policy such as vaccine schedules, and exploring potential epidemiological mechanisms for vaccine failure. Due to the high frequency of sub-clinical infections and transient faecal viral shedding, the true incidence of rotavirus infection is hard to estimate outside of large closely monitored cohort studies, which are logistically challenging and expensive to conduct. In this context, sero-surveys may represent a pragmatic alternative. Serum anti-rotavirus immunoglobulin A (IgA) develops in response to rotavirus infection, reflects intestinal IgA, which is thought to be key in long term protection against rotavirus, and has been shown to correlate well with protection against severe rotavirus disease[19–23]. Previously heterogenicities in sampling methods and laboratory protocols meant that comparison of IgA responses between populations was challenging. Recently however clinical vaccine trials have given rise to more standardised methods, facilitating comparisons of sero-response to vaccine and pre-vaccine exposure to rotavirus across settings [24,25]. To our knowledge this has not been utilised to compare incidence of rotavirus infection between populations although serological data has previously been used to evaluate prevalence and incidence of other pathogens including human papilloma virus and Campylobacter[26,27]. Interpretation of serological data can be complicated, however, by natural fluctuations and measurement errors that mask or mimic infection and traditional methods of analysis of serological data such as pre-defined cut-offs in levels and/or “fold increase” can be prone to misclassification[27,28].

This study used mixture models as an analytical approach to estimate incidence of rotavirus infection using serum anti-rotavirus IgA titres in two different low-income, unvaccinated populations: an urban setting in Vellore, Southern India, and a rural setting in Karonga, Northern Malawi. Our mixture models specify mixtures of two (“positive” and “negative”) component gaussian distributions, and may offer an advantage over more traditional methods of evaluating serological data as they evaluate data probabilistically. Models were used to describe patterns of exposure over the first 3 years of life in Vellore and to compare incidence rates between infants in Vellore and Karonga in order to increase understanding of force of rotavirus infection in young children from different LIC prior to rotavirus vaccine introduction.

## Methods

### Study population and sample collection

This study utilised serum samples collected from young children in two distinct locations; an urban slum setting in Vellore, Southern India; and Karonga, a rural setting in Northern Malawi. In Vellore, serum samples were collected from 373 children enrolled in a birth cohort designed to evaluate natural immunity to rotavirus infection. Data from this cohort were collected between 2002 and 2006. Samples for serology were collected at 6 month intervals from birth and biweekly stool samples were collected for three years onwards. Stool samples were tested for rotavirus and rotavirus infection defined as positive on two enzyme-linked immunosorbent assay (ELISA) tests or reverse-transcriptase–polymerase-chain-reaction (RT-PCR). A detailed description of this cohort has been published[29]. In Karonga, serum samples were collected at 6, 26 and 52 weeks of life from 190 children born in the Karonga Health and Demographic Surveillance System between November 2008 and November 2010[30]. These infants were enrolled as part of a birth cohort investigating pneumococcal carriage in Human Immunodeficiency Virus (HIV) exposed mothers and their infants, and approximately 28% of the infants were exposed to HIV[31]. A total of 112 of these 190 children had complete sets of three serum samples. Samples were selected for IgA analysis if they contained more than 100μl of serum, resulting in 198 samples from 66 children. Neither population had routine rotavirus vaccine introduced at the time of specimen collection. Anonymised serological data from Vellore and Karonga can be found in S1 File in the supplementary materials (S1A and S1B File respectively). Written consent for infant participation in each study was obtained from the responsible parent or guardian, and this process was approved by the respective ethics committees (Christian Medical College, Vellore and National Health Sciences Research Committee in Malawi).

### Laboratory methods

Anti-rotavirus IgA antibodies were measured using a standard sandwich ELISA [32]. Methods, standards and controls used were the same in both sites, with the exception that Vellore used 2 x 10 fold dilution of sera while Karonga used 4 x 2 fold dilutions. Briefly, 96 well plates coated with rabbit anti-rotavirus hyperimmune serum were incubated with WC3 rotavirus containing cell culture lysate (MA104). Sera prepared in respective dilutions in blocking solution (1% blotto) were added to the plate. Anti-rotavirus IgA detection was performed using biotinylated rabbit anti-human IgA (Jackson ImmunoResearch Lab, USA) an avidin-biotin-peroxidase complex (Vecastain ABC kit; Vector) and a peroxidase substrate (o-Phenylenediaminedihydrochloride; Sigma) and H2O2 (Sigma). Rotavirus-specific IgA titres were quantitated against a standard curve (serial 2 fold dilution of control plasma calibrated against an international standard) and positive, negative and uninfected cell lysate controls were added to each plate.

Results were expressed as mean adjusted titres of at least two values per serum with a coefficient of variation < 20%. Positive samples in which the titres obtained in 2 or more dilutions had >20% coefficient of variation in two repeat tests were expressed using trimmed geometric means. Results which fell below the lower limit of quantification defined by the standard curve were defined as below the limit of detection and were recorded as zero. Results above the upper limit of detection were repeated with serial dilutions until a quantifiable result was obtained. Quantitative values for clinical test samples were expressed as IU/ ml IgA.

### Statistical analysis

Analysis comprised 4 stages: i) descriptive analysis; ii) estimation of risk of seroconversion in the first 3 years of life in Vellore and thereby derivation of incidence; iii) comparison of risk and incidence estimates in Vellore and Karonga, and iv) calculation of antibody decay rate.

We compared independent and paired continuous variables using sign-rank and rank sum tests respectively, and chi-squared tests to compare independent proportions. For this analysis seroconversion was defined as titres ≥20IU/ml[33].

The Vellore dataset was then used to investigate the pattern of rotavirus exposure in the first 3 years of life. Two component Gaussian mixture models, one component assumed to correspond to uninfected individuals and one, with larger values, to seroconverted (presumed infected) individuals, were used to estimate the risk of seroconversion at 6 monthly time intervals, where the risk refers to the proportion (prevalence) of samples assigned to the positive or “infected” distribution[26,27]. Antibody titres were log transformed after adding one to the value of each titre to allow log transformation of zero values, and models were fit to the increment in log transformed titres between each of the time points (i.e. between 6 and 26 weeks [d1], 26 and 52 weeks [d2], 52 and 78 weeks [d3], 78 and 104 weeks [d4], 104 and 130 weeks [d5] and 130 and 156 weeks [d6]). There were large numbers of zero values (representing no change in antibody titre) for each time point. These did not fit a Gaussian distribution, and were therefore excluded from fitting the models. As these values clearly represented no evidence of re-infection, they were added back into the uninfected component for calculations of risk and incidence. Bootstrap confidence bounds were calculated.

Rotavirus infection incidence λ, during the interval τ between each time point was calculated based on the relationship between the risk and incidence rate using the formula below, where *p* corresponds to the bootstrap estimate of mean risk of sero-conversion[34]:

$$\lambda =\frac{-ln(1-p)}{\tau }$$

The same methods were then used to estimate the risk and incidence of seroconversion in Karonga between 6 and 26, 26 and 52 and 6 and 52 weeks of life in order to compare exposure to rotavirus infection in infancy between the Vellore and Karonga populations. The timing of the first sample differed between populations (6 weeks of age in Karonga vs birth in Vellore), however for the purposes of this analysis this baseline time point was assumed to be the same. For additional validation, and to investigate if the pattern of increment differed significantly between populations we calculated the difference between increments in each location by subtracting [d2] from [d1] and compared the mean value obtained between locations using a two-sample t-test.

To evaluate the use of mixture models, seroconversion was also calculated using two alternative standard definitions; fold increase and a pre-defined cut-off of anti-rotavirus IgA titres ≥ 20IU. For calculation of fold increase 0.1 was added to each assay result (to allow calculation of fold increase for zero values), and seroconversion was defined as a three-fold or greater rise between time points. For the cut-off of ≥20IU, sero-positivity was defined as IgA titres ≥ 20IU and becoming seropositive between time points was considered seroconversion.

Finally, in order to evaluate the likelihood of capturing repeated infection episodes using mixture models, a decay rate was calculated for anti-rotavirus IgA using a subset of children from the Vellore dataset. 87 children were identified who had a stool or serologically confirmed rotavirus infection in the first 26 weeks of life, and no evidence of re-infection between 26 and 52 weeks. Antibody decay was calculated based on the log of the fold increase in titres between 26 and 52 weeks, where any value less than one indicates a decline in titre. Anonymised serological data used to estimate decay rate can be found in S1 File in the supplementary materials (S1 File).

Statistical analyses were conducted using Stata 12 (StataCorp, USA), GraphPad Prism 6 (GraphPad Software Inc, USA), and R 3.0.2 (R Foundation for Statistical Computing, Austria).

## Results

### Descriptive analysis

**Fig 1:**
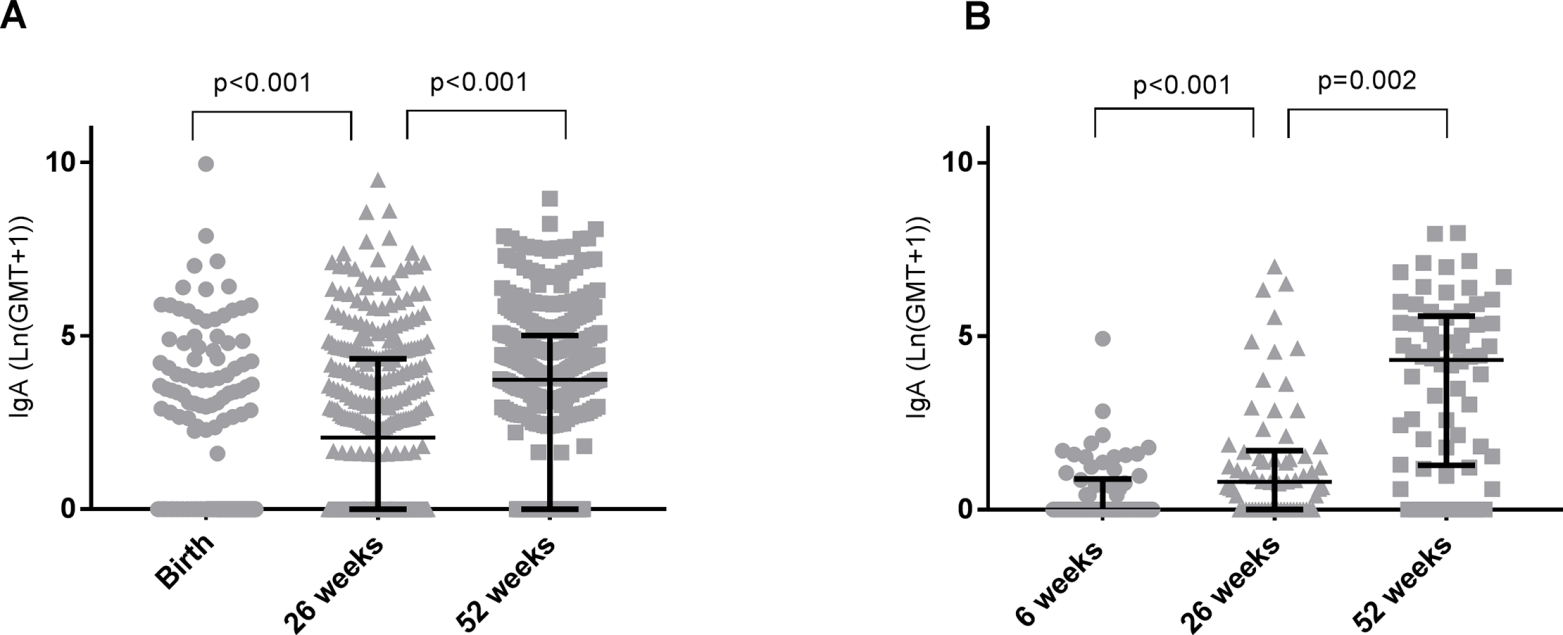
**Increase in IgA titres over time in Vellore (A) and Karonga (B).** Error bars represent median and IQR. P values represent sign-rank tests for paired medians.

IgA titres rose incrementally in both Vellore and Karonga, with significant rises in median IgA titres between time points (Fig 1). The proportion of children with anti-rotavirus IgA titres ≥20IU/ml was greater in Vellore than Karonga at 6 (15.43% vs 1.52%, chi-squared test p = 0.002) and 26 (37.78% vs 13.64%, chi-squared p<0.001) weeks of life, but there was no significant difference between the two populations at 52 weeks of life (61.13% vs 60.61%, chi squared p = 0.937).

### Patterns of rotavirus infection in first 3 years of life

**Fig 2:**
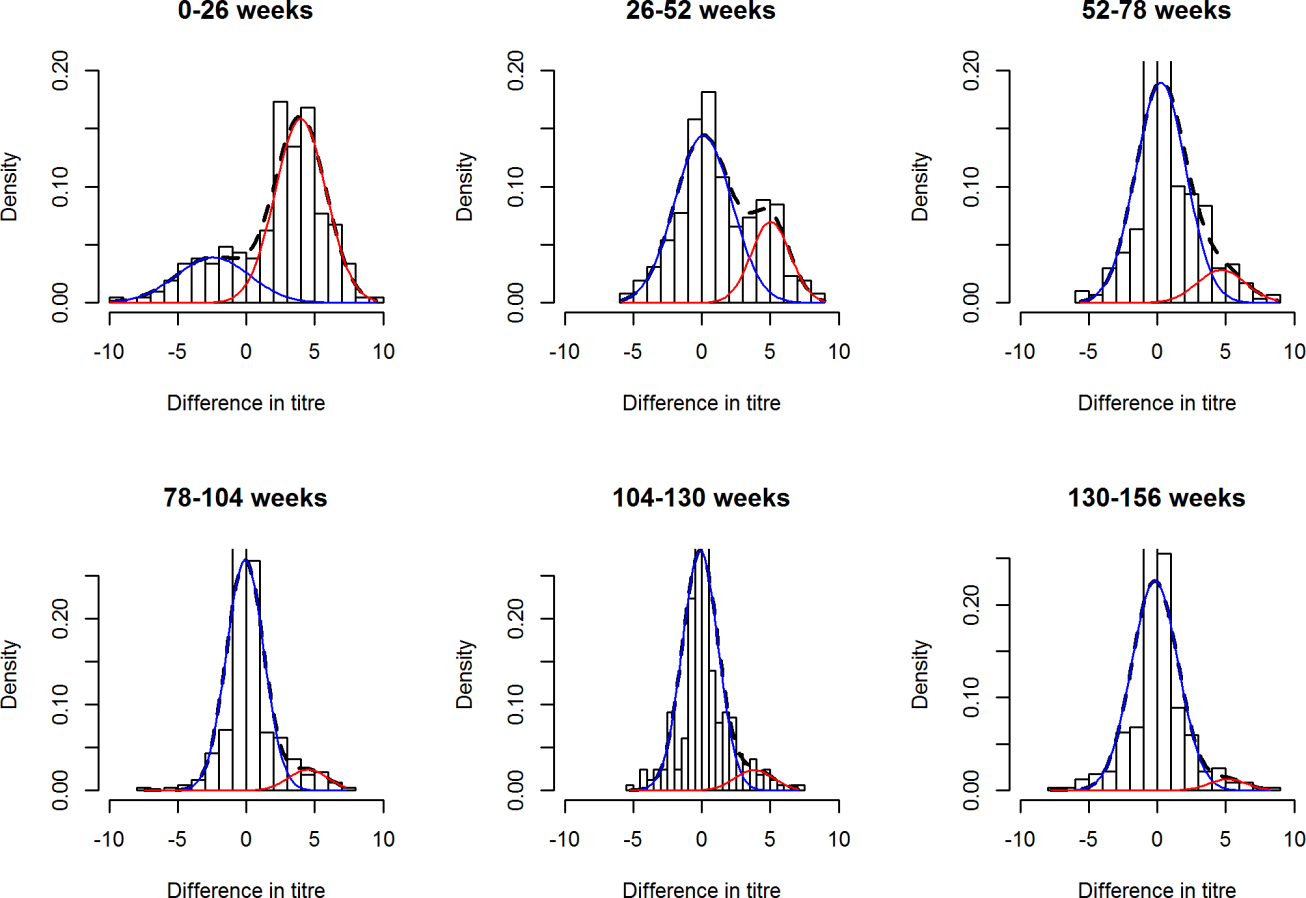
Output from mixture models showing positive (“infected”) and negative (“uninfected”) distributions for increment in log transformed anti-rotavirus IgA titres between 6 month time points in Vellore. The dashed line represents the (fitted) mixture distribution, the red line the constituent distribution for the “infected” and the blue line the constituent distribution for the “uninfected”. Bars represent values for difference between titres.

Mixture models fit to the Vellore data set over 3 years showed an initial high frequency of rotavirus infection, with a risk of seroconversion of 0.41 (95% confidence interval [CI]: 0.27, 0.56) between birth and 6 months, which declined with each subsequent time interval (Fig 2 and Table 1).

**Table 1:** Parameter estimates from mixture models for rotavirus infection in Vellore and Karonga.

	Mean 1*	SD* 1	Mean 2	SD 2	Risk**	95% CI	Incidence^Ϯ^	95% CI
Vellore								
**0–26 wks**	-2.44	2.68	3.97	1.86	0.41	0.27,0.56	1.05	0.64, 1.64
**26–52 wks**	0.11	2.08	5.02	1.42	0.20	0.08,0.40	0.44	0.17, 1.02
**52–78 wks**	0.26	1.84	4.69	1.77	0.18	0.02,0.72	0.39	0.04, 2.57
**78–104 wks**	-0.08	1.36	4.52	1.43	0.13	0.02,0.54	0.29	0.04, 1.57
**104-130wks**	-0.12	1.31	3.84	1.41	0.11	0.02,0.47	0.24	0.04, 1.29
**130–156 wks**	-0.17	1.69	5.14	1.36	0.05	0.00,0.78	0.10	0.00, 3.04
Karonga								
**6–26 wks**	0.40	1.45	4.87	1.22	0.15	0.04,0.44	0.34	0.08, 1.17
**26–52 wks**	0.08	1.31	4.48	1.35	0.50	0.33,0.68	1.41	0.79, 2.29
Increment over first year of life								
**Vellore**								
**0–52 wks**	-0.35	2.62	4.62	1.53	0.55	0.48,0.62	0.80	0.65, 0.97
**Karonga**								
**6–52 wks**	-0.22	0.59	4.26	1.82	0.71	0.42,0.90	1.25	0.54, 2.28

Data from Vellore for 156 weeks, from Karonga for 52 weeks.

*Where mean 1 and SD1 refer to mean and standard deviation (SD) for distribution 1 (increment in log transformed titres for uninfected children), and mean 2 and SD2 to mean and standard deviation for distribution 2 (increment in log transformed titres for infected children).

**Mean risk of seroconversion and confidence intervals (CI) derived from bootstrap estimates

^Ϯ^ Incidence rate of rotavirus infection derived from mean risk using formula stated previously. Incidence rate in episodes per child year.

### Comparison of patterns of rotavirus infection in first year of life between Vellore and Karonga

**Fig 3:**
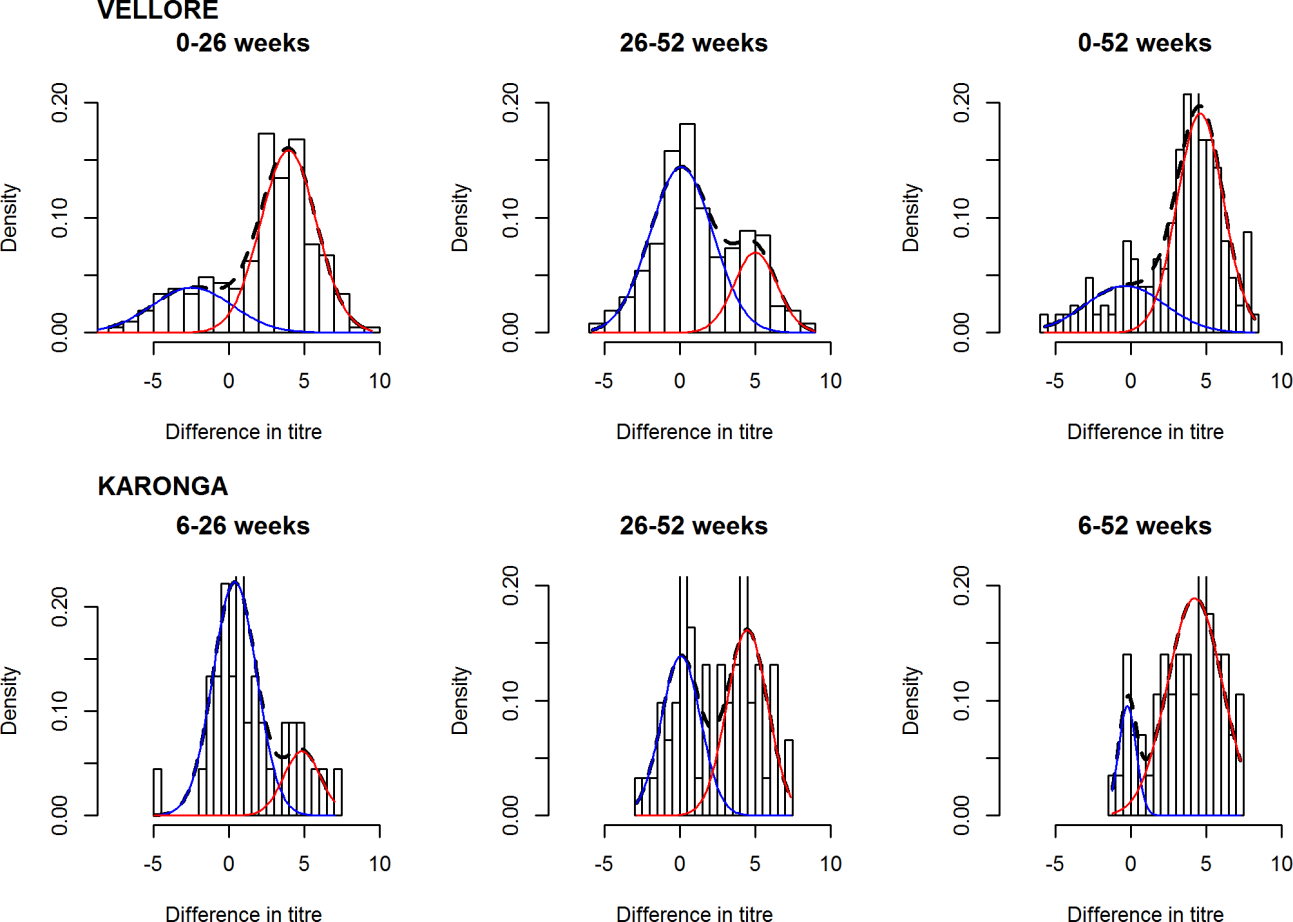
Output from mixture models showing positive (“infected”) and negative (“uninfected”) distributions for increment in log transformed anti-rotavirus IgA titres for the first year of life in Karonga and Vellore. The dashed line represents the (fitted) mixture distribution, the red line the constituent distribution for the “infected” and the blue line the constituent distribution for the “uninfected”.

Fitting mixture models to the Karonga dataset demonstrated that incidence of rotavirus infection varied by time and between the populations. Between 6 weeks and 26 weeks incidence of infection in Karonga was lower than observed in Vellore with 0.34 episodes/child year (95% CI: 0.08, 1.17) compared to 1.05 episodes/child year (95% CI: 0.64, 1.64) (Fig 3 and Table 1). In comparison, incidence was considerably higher in Karonga between 26 and 52 weeks than in Vellore (1.41 episodes/child year [95% CI 0.79, 2.29] vs 0.44 episodes/child year [95% CI: 0.17, 1.02]) (Fig 3 and Table 1). There was no clear difference between the two populations when incidence was calculated between 6 and 52 weeks (1.25 episodes per child year [95% CI 0.54, 2.28] in Karonga, versus 0.80 [95% CI: 0.65, 0.97] in Vellore).

The mean difference in titres ([d2]-[d1]) was significantly smaller in Vellore than Karonga (-0.35 in Vellore and 1.45 in Karonga, two sample t-test p = 0.004). This is likely due to the high risk of seroconversion in the first six months of life and subsequent lower relative increase in titres in the second six months of life in Vellore.

Overall, estimates of incidence using the mixture models were similar to the two alternative definitions of seroconversion (Table 2). The notable exception was for estimating incidence between 6 and 26 weeks, when using fold increase resulted in a substantially higher estimate in Karonga (1.10 episodes/child year [95% CI: 0.74, 1.59]) than using mixture models or IgA titres > = 20 IU (0.34 episodes/child year [95%: CI 0.08, 1.17] and 0.29 episodes/child year [95% CI: 0.15, 0.56], respectively). Apart from this, all three methods showed higher incidence of infection in Vellore compared to Karonga in the first 6 months of life, and higher incidence in Karonga compared to Vellore between 26 and 52 weeks.

**Table 2:** Risk of seroconversion and incidence of rotavirus infection between 0/6 and 26 weeks, 26 and 52 and 0/6 and 52 weeks for Vellore and Karonga using mixture models, fold increase and IgA titres> = 20IU.

		Mixture Model		Fold Increase		IgA titres> = 20IU	
		Risk*	Incidence ^Ϯ^	Risk	Incidence ^Ϯ^	Risk	Incidence ^Ϯ^
0/6 weeks to 26 weeks							
Vellore	Estimate	0.41	1.05	0.44	1.16	0.31	0.73
	95% CI	0.27,0.56	0.64,1.64	0.39,0.49	0.98,1.36	0.26,0.36	0.60,0.88
Karonga	Estimate	0.15	0.34	0.42	1.10	0.14	0.29
	95% CI	0.04,0.44	0.08,1.17	0.31,0.55	0.74,1.59	0.07,0.26	0.15,0.56
26 to 52 weeks							
Vellore	Estimate	0.20	0.44	0.35	0.87	0.28	0.66
	95% CI	0.08,0.40	0.17,1.02	0.30,0.41	0.73,1.04	0.24,0.33	0.54,0.80
Karonga	Estimate	0.50	1.41	0.61	1.86	0.50	1.39
	95% CI	0.33,0.68	0.79,2.29	0.48,0.72	1.31,2.54	0.38,0.62	0.95,1.94
0/6 weeks to 52 weeks							
Vellore	Estimate	0.55	0.80	0.60	0.91	0.50	0.70
	95% CI	0.48–0.62	0.65–0.97	0.54–0.65	0.78–1.05	0.45–0.56	0.60–0.82
Karonga	Estimate	0.71	1.25	0.74	1.36	0.59	0.89
	95% CI	0.42–0.90	0.54–2.28	0.62–0.84	0.97–1.80	0.47–0.71	0.63–1.22

*Mean risk and confidence intervals (CI) derived from bootstrap estimates

^Ϯ^ Incidence rate derived from risk estimate using formula stated previously. Incidence rate in episodes per child year.

### Anti-rotavirus IgA antibody decay

**Fig 4:**
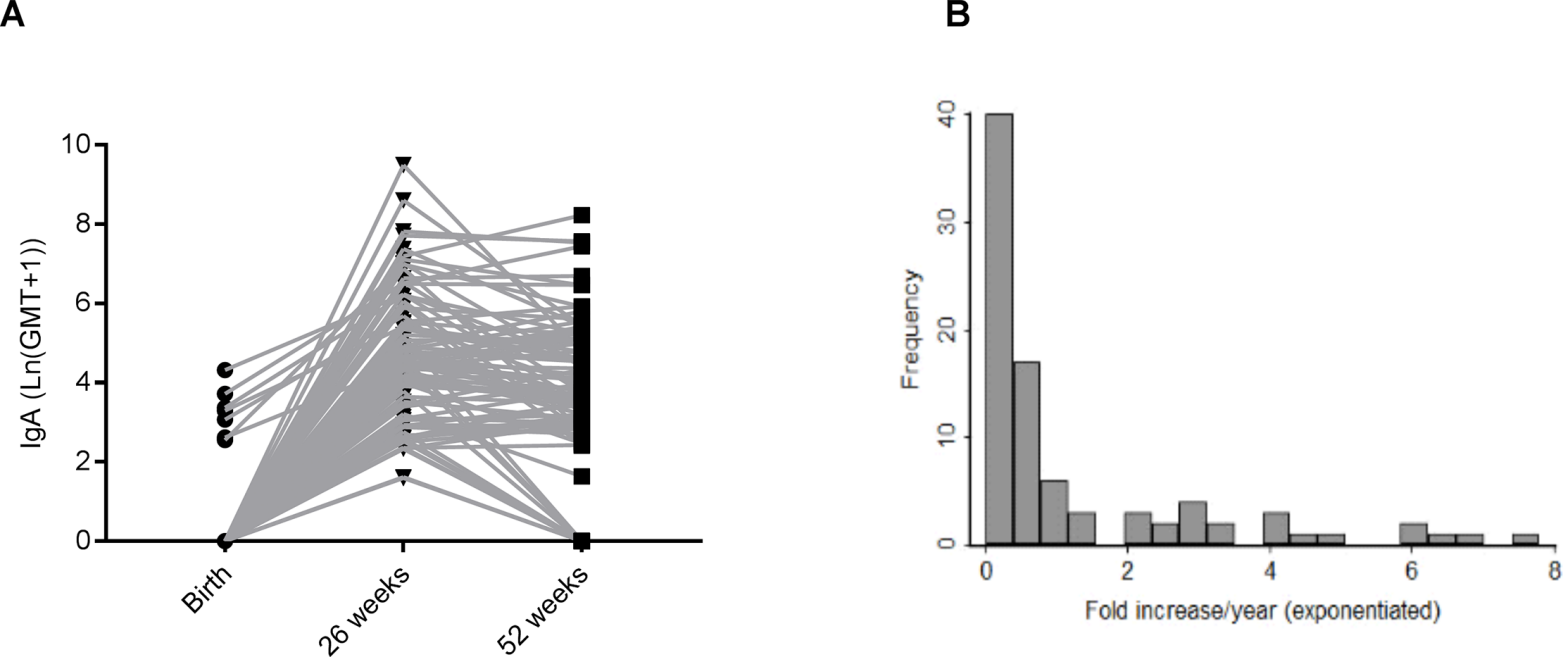
Anti-rotavirus IgA decay rates. A) Log transformed titres at 0, 26 and 52 weeks for 87 children included in IgA decay analysis. B) Fold increase in titres per year for the same children. Titres log transformed then exponentiated.

Based on the log of fold increase, anti-rotavirus IgA titres showed a relatively rapid decay with a mean fold increase of 0.09 fold/year, which is equivalent to a > 10X reduction in antibody titres) following an initial infection (Fig 4).

## Discussion

Identifying heterogeneities in infant rotavirus transmission patterns is important as force of infection may be a contributing factor to the reduced vaccine effectiveness observed in LIC, and understanding patterns of incidence may help inform vaccine scheduling and improve vaccine impact. This study demonstrates notable differences in force of infection between infants from Vellore, India, and Karonga, Malawi in the first year of life, with children in Vellore seemingly infected earlier in life than those in Karonga.

Although the confidence intervals for risk and incidence estimates are wide and overlap considerably, the validity of these findings are corroborated by the observation that the same patterns are seen when a pre-defined cut-off (≥20 IU) is used to define seroconversion, and that a significant difference is observed in mean titre increment from the first six to the second six months of life between populations. One explanation for the observed difference in exposure patterns is variation in population dynamics between the two study sites; Vellore is an urban slum environment with a population density of 17,000/km^2^[35] in contrast to the rural Karonga district, with a population density of approximately 264/km^2^[30]. In support of this, crowding has previously been associated with increased frequency of rotavirus infection[36], and a recent study from Dhaka, Bangladesh demonstrated that rotavirus incidence in the densely populated core of the city was 3 times greater than in the more sparsely populated periphery [37].

Using anti-rotavirus IgA titres to assess force of rotavirus infection is attractive, particularly as in recent years IgA titres in infants have been measured in a standardised manner across numerous different populations as part of vaccine efficacy trials, making comparisons between studies and sites easier and resulting in banks of data which could be utilised to understand global trends in population level rotavirus incidence[38]. Mixture models offer an advantage over traditional methods of defining seroconversion, as they provide a visual interpretation of the data, and evaluate infection probabilistically (i.e. the probability of each sample falling into the positive or negative distribution), and thus avoid the assumption of an, somewhat arbitrary, absolute cut off. Uncertainty around estimates can be evaluated using boot-strapping and expressed as confidence intervals. The ability to estimate rotavirus incidence with relative ease could increase understanding of differences in patterns of exposure between populations. In this study, the risk of seroconversion identified using mixture models is reasonably consistent with that identified using the more traditional methods of a defined cut-off, or fold increase from baseline. The exception to this is risk of seroconversion defined by fold increase in the first six months of life in Karonga, which identified significantly more children as having seroconverted that either mixture models or a pre-defined cut-off. One explanation for this could be mathematical artefact due the lower proportion of children who had IgA titres ≥20 IU at the time of first sample in Karonga[28].

Widespread introduction of rotavirus vaccination has had a substantial impact on the burden of diarrhoeal disease in children from the poorest countries[39–41]. With the success of programmatic vaccine introduction and encouraging but sub-optimal vaccine effectiveness reported from LIC it is important to target vaccine failure and residual rotavirus disease. High force of infection may lead to higher trans-placental maternal IgG and anti-rotavirus IgA titres in breast milk, both of which may impact on vaccine response in infants[42]. An additional dose of vaccine or delayed vaccine schedule such as recently trialled in Ghana may improve vaccine response in this instance[43]. Understanding the timing of peak rotavirus incidence may also inform decisions around optimal vaccine schedules; for example a high burden of very early disease such as observed in Vellore could lead to consideration of a neonatal dose of vaccine, such as the candidate vaccine RV3-BB, currently undergoing immunogenicity trials[44]. The variation in transmission patterns between two different low income settings identified in this study suggests that different populations may require different approaches. It is intriguing that the model derived mean increment for Vellore in the first 6 months of life is a negative value. This is unlikely to reflect maternal antibody as this is usually IgG, which is not measured by this study. One possibility could be a number of infants with neonatal infection and subsequent sero-response, which then declines substantially over the next 6 months.

Whilst serum IgA is possibly the best currently available marker of recent rotavirus infection, it is not a perfect correlate of protection against rotavirus[45]. Using serology alone to estimate rotavirus incidence will under-estimate the true burden; cohort studies from Vellore and Mexico collected stool and serum samples to estimate rotavirus incidence and in both approximately 25% of infections were identified in stool alone without a corresponding rise in antibody titres [29,46]. The degree of under-estimation seems unlikely to vary considerably across populations however, and broad patterns in rates of infection across should still be comparable. It is also possible that sero-response only captures first infection; that subsequent infections may not boost IgA levels sufficiently for re-infection to be captured. However the rapid decay in IgA titres (~10 fold per year) following an initial infection seen in this study argues against this.

This analysis of existing data included a small number of children, particularly from Karonga, which likely contributes to wide confidence bounds around risk and incidence estimates. Those children from Karonga included in the study are a relatively small proportion of available children (66/190, 35%). This was due to available volume of serum and reflects the challenges of obtaining blood samples from young children. While this will have contributed to the small sample size it is unlikely that children with larger sample volumes differ systematically from those with smaller sample volumes and should not affect the representative nature of the results. Timing of collection of the first serum sample differed by site, but there were very few infections identified in either cohort before the first sample therefore this is unlikely to have affected the comparison. Approximately 28% of infants from Karonga were HIV exposed, however as HIV infected infants have comparable IgA responses to those of HIV uninfected infants following rotavirus vaccine and rotavirus does not seem to be more frequent in HIV infected children[47–49], it seems unlikely that HIV exposure status should substantially influence IgA responses to natural rotavirus infection.

## Conclusions

Fitting mixture models to anti-rotavirus serum IgA titres is an efficient and inexpensive quantitative method to estimate population incidence in young infants. Using these models we identified that infants in Vellore are infected with rotavirus at a younger age than those in Karonga. Identifying heterogeneities in transmission such as these may help increase understanding of mechanisms behind reduced vaccine effectiveness and inform vaccine scheduling to optimise protection for infants against rotavirus. This is crucial given the observed lower vaccine effectiveness and ongoing burden of disease in low income settings.

## Supporting information

S1 File Serological data for Karonga and Vellore. This consists of 3 datasets; S1A containing data IgA data from Vellore, S1B containing IgA data from Karonga, and S1C containing a subset of the Vellore data used to estimate IgA decay rate.
(ZIP)
